# Microstructure and Tensile Properties of Friction Stir Processed Mg–Sn–Zn Alloy

**DOI:** 10.3390/ma11040645

**Published:** 2018-04-23

**Authors:** Xiaoyang Chen, Qiao Dai, Xingcheng Li, Yalin Lu, Yang Zhang

**Affiliations:** Key Lab of Advanced Material Design and Additive Manufacturing of Jiangsu Province, Jiangsu University of Technology, Changzhou 213001, China; cxy@jsut.edu.cn (X.C.); daiqiao@jsut.edu.cn (Q.D.); sgylxc@jsut.edu.cn (X.L.); jxlyl@jsut.edu.cn (Y.L.)

**Keywords:** Mg–Sn–Zn alloy, friction stir processing, microstructure, texture, tensile properties

## Abstract

In this study, as-cast Mg–6Sn–2Zn (wt.%) alloy was subjected to friction stir processing (FSP) and the microstructure and tensile properties of FSP Mg–6Sn–2Zn samples were investigated. It was found that, in the stir zone (SZ) of FSP Mg–6Sn–2Zn samples, α-Mg grains were significantly refined via dynamic recrystallization (DRX) and the Mg_2_Sn phase was broken and partially dissolved. The microstructure in SZ was nonuniform and DRXed grains in the SZ-up regions were coarser than those in the SZ-down regions. Coarse broken Mg_2_Sn particles were observed in the SZ-up regions, while only fine Mg_2_Sn particles were observed in the SZ-down regions. Strong {0001} basal texture developed in the SZ regions of Mg–6Sn–2Zn samples after FSP. The increase of travel speed had little effect on the texture of different SZ regions. The ductility of FSP Mg–6Sn–2Zn samples was obviously improved, while the improvement in strength was negligible when compared to the as-cast sample. The tensile properties of FSP Mg–6Sn–2Zn samples were influenced by grain refinement, texture modification, and the breaking up and dissolution of the Mg_2_Sn phase.

## 1. Introduction

Mg alloys are among the lightest metallic structural materials, and possess salient advantages including high specific strength and stiffness, good thermal conductivity, and good damping performance, etc. In recent years, Mg alloys have shown broad prospects in areas of aerospace, automobile, and communication industries [1,2]. Nevertheless, the large-scale application of Mg alloys is still limited by their inherent imperfections, especially their poor heat resistance [3]. Mg–Sn alloys are considered as one kind of promising heat-resistant Mg alloy with a low cost, since the second phase in Mg–Sn alloy, i.e., the Mg_2_Sn phase, has a melting point close to the Mg–rare earth (RE) phase and the diffusion of Sn atoms in the Mg matrix is difficult to achieve [4,5]. Therefore, Mg–Sn alloys are a probable substitute for traditional expensive heat-resistant Mg–RE alloys.

To date, the majority of Mg alloy parts have been produced by the casting process [6]. Coarse grains and defects (including segregation and porosity) restrict the comprehensive mechanical behavior of Mg alloy castings. Besides, due to the hexagonal close-packed (HCP) structure of pure Mg, the ductility of cast Mg alloys is sometimes unsatisfying. Grain refinement is generally believed effective in improving the comprehensive mechanical behavior of metallic materials. Severe plastic deformation (SPD) is a widely-concerned kind of grain refinement methods. Metallic materials are broken and refined by exerting a large force or strain rate during the SPD process. The mechanical behavior of metallic materials is usually improved obviously after the SPD process [7]. Friction stir processing (FSP) is a newly-developed SPD process that is based on friction stir welding (FSW) [8,9]. During FSP, plastic deformation, mixing, breaking, and heat input are induced by the high-speed rotation of a stirring pin, simultaneously resulting in grain refinement, homogenization, and densification [10,11].

In recent years, FSP has been used to modify the microstructure and improve the mechanical behavior of several cast Mg alloys [12,13]. For example, Wang et al. reported that FSP caused a remarkable grain refinement, homogenization, and breaking up and dissolution of the β-Mg_17_Al_12_ phase in cast AZ31 alloy [14]. Xiao et al. found that, after FSP, the grains of a Mg–Gd–Y–Zr alloy were refined, the coarse Mg_5_(Gd,Y) phase was dissolved, and both the strength and ductility were improved remarkably [15]. Based on existing studies on FSP of cast Mg alloys, it is reasonable to extrapolate that FSP is also able to improve the mechanical behavior of cast Mg–Sn alloys due to the significant grain refinement. However, there are few reports available on FSP of Mg–Sn cast alloys so far.

In order to achieve the desired effect of solution and precipitation strengthening, the Sn element content was often chosen as 5–7 wt.% [16,17]. It was also found that the addition of Zn element could enhance the ductility and precipitation strengthening of Mg–Sn based alloys [18]. Therefore, in this study, a Mg–6Sn–2Zn (wt.%) alloy was prepared by gravity casting. The as-cast alloy was then subjected to FSP with different travel speeds. The effect of the travel speed on the microstructure and tensile properties of FSP Mg–6Sn–2Zn samples was investigated. The mechanism of microstructure evolution and the influence factors of mechanical properties of FSP Mg–6Sn–2Zn samples were also discussed.

## 2. Materials and Methods 

**Figure 1:**
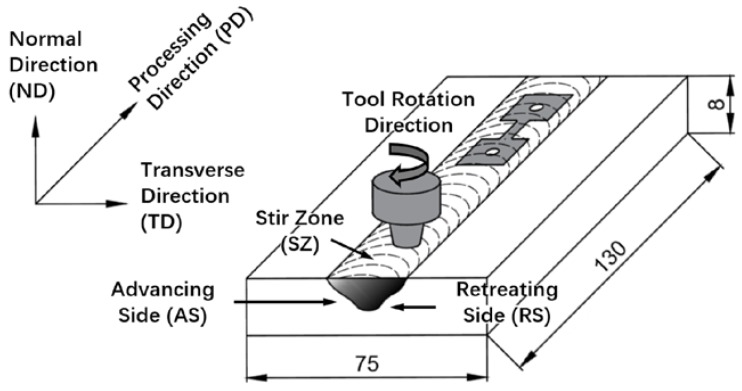
The schematic view of friction stir processing (FSP) in this study.

As-cast Mg–6Sn–2Zn ingots were prepared with commercial pure Mg (99.95 wt.%), Sn (99.99 wt.%), and Zn (99.99 wt.%). The raw materials were melted in a resistance crucible furnace under a protection gas of mixed SF_6_ and CO_2_ (1:100, vol.). Pure Mg was melted first, and then pure Sn and Zn were added at 730 °C. Hand stirring was conducted for 3 min to assure the homogeneous distribution of the alloying elements. After holding at 730 °C for 10 min, the melt was poured into a permanent mold that had been preheated to 200 °C. As-cast Mg–6Sn–2Zn ingots were used as the base metal (BM). Plates with dimensions of 130 mm × 75 mm × 8 mm were cut from the as-cast ingots for FSP. The surfaces of the plates were ground manually to remove any oxides. FSP was conducted on an FSW machine (FSW-LM-BM16, FSW Technology Co., Ltd., Beijing, China). The tool had a shoulder 16 mm in diameter and a pin 6 mm in length, 3.5 mm in tip diameter, and 5.5 mm in root diameter. One-pass FSP was performed for each sample. The rotation speed was set as 1200 rpm and the travel speeds were set as 60, 90, and 120 mm/min. Figure 1 shows the schematic view of FSP.

Specimens for microstructure characterization were cut in a transverse direction (TD)-normal direction (ND) plane. Microstructure characterization was conducted by X-ray diffraction (XRD) (Panalytical, XPERT POWDER, Almelo, The Netherlands), optical microscope (OM) (Zeiss, Primotech, Berlin, Germany), and scanning electron microscope (SEM) (Zeiss, Simga 500, Oberkochen, Germany). Quantitative metallurgical analysis was carried out by quantitative image analysis software image pro plus 5.0 (Media Cybernetics, Rockville, MD, USA). Texture characterization was conducted by electron backscattered diffraction (EBSD) (Oxford Instruments, Oxford, UK). Specimens for the tensile test with a gage size of 10 mm in length, 3.5 mm in width, and 1.5 mm in thickness were cut from as-cast and FSP Mg–6Sn–2Zn alloys. The tensile specimens from the FSP Mg–6Sn–2Zn alloy were cut along the processing direction (PD) with the gage in the stir zone (SZ), which is also shown in Figure 1. The tensile test was performed using a universal testing machine (CMT-5205, WANCE Co., Ltd., Shenzhen, China) with a strain rate of 1.67 × 10^−3^ s^−1^.

## 3. Results

**Figure 2:**
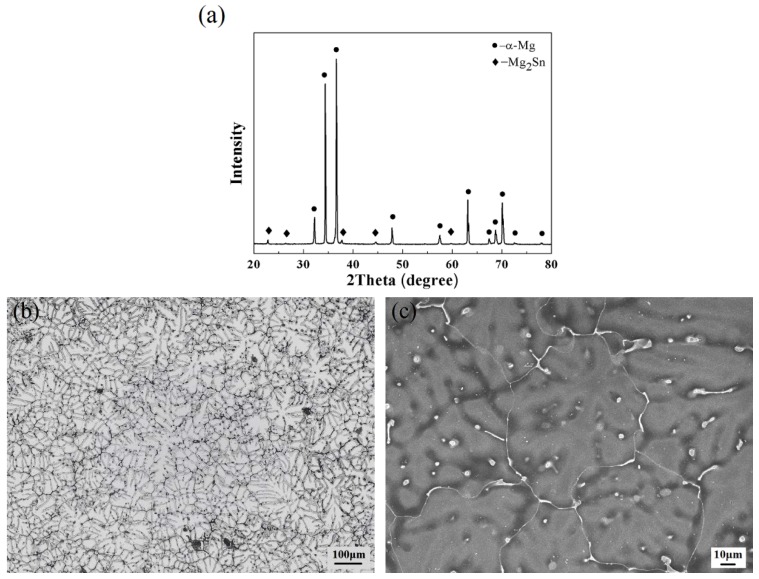
XRD analysis result and microstructure of the as-cast Mg–6Sn–2Zn alloy, which was used as the base metal (BM) in this study: (**a**) XRD analysis result; (**b**) optical microstructure; and (**c**) SEM image.

Figure 2 shows the XRD analysis result and microstructure of the as-cast Mg–6Sn–2Zn alloy, which was used as the base metal (BM) in this study. According to the XRD result in Figure 2a, the as-cast Mg–6Sn–2Zn alloy consisted of α-Mg and Mg_2_Sn phases. The XRD result indicates that the addition of Zn element does not lead to the formation of any Zn-containing phase. As shown in Figure 2b,c, the Mg–6Sn–2Zn alloy exhibited a typical as-cast microstructure with a coarse dendritic α-Mg phase and a eutectic Mg_2_Sn phase. The coarse Mg_2_Sn phase was mainly distributed at the grain boundaries.

**Figure 3:**
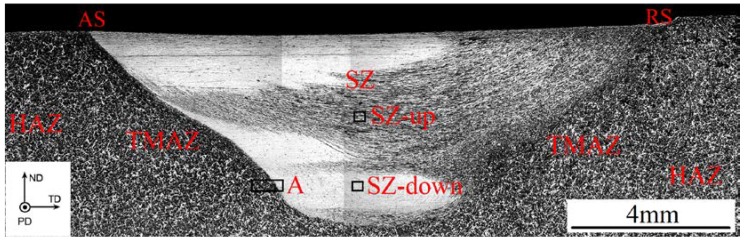
Optical macrostructure of a cross-section of the FSP Mg–6Sn–2Zn sample at the travel speed of 60 mm/min.

Figure 3 shows the cross-section optical macrostructure of the representative FSP Mg–6Sn–2Zn sample (with a travel speed of 60 mm/min). Since the observation reveals that there was little different between the macrostructural characteristics of the three FSP samples, the cross-section optical macrostructure of the other two samples was omitted. As seen in Figure 3, after FSP, a basin-shaped nugget is formed in every sample. The left side is the advancing side (AS) and the right side is the retreating side (RS). Three macrostructural zones induced by FSP are apparent, i.e., the stir zone (SZ), the thermo-mechanically affected zone (TMAZ), and the heat-affected zone (HAZ). The microstructure transition from the TMAZ to the SZ on the AS is sharp, while it is smooth on the RS. There is a clear line of demarcation between the TMAZ and SZ on the AS, while the line of demarcation between the TMAZ and SZ on the RS is blurred. It is also observed from the optical macrostructure that the microstructure in the SZ of the FSP Mg–6Sn–2Zn sample is nonuniform. In order to characterize the microstructure of the SZ in greater detail, two representative regions, i.e., SZ-up and SZ-down, were selected from the cross-section of the SZ, which were located at the center line (seen in Figure 3). SZ-up is about 2 mm down from the top surface and SZ-down is about 4 mm down from the top surface. For each FSP sample, the characterized regions are kept the same.

**Figure 4:**
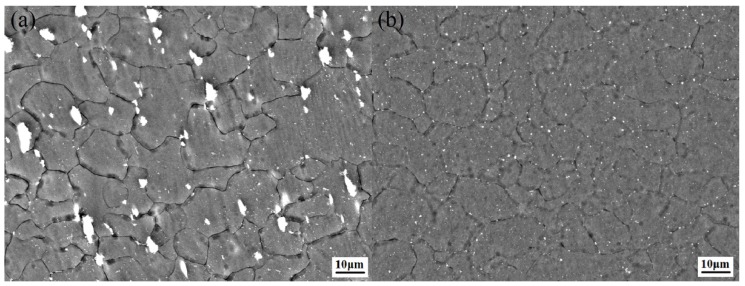

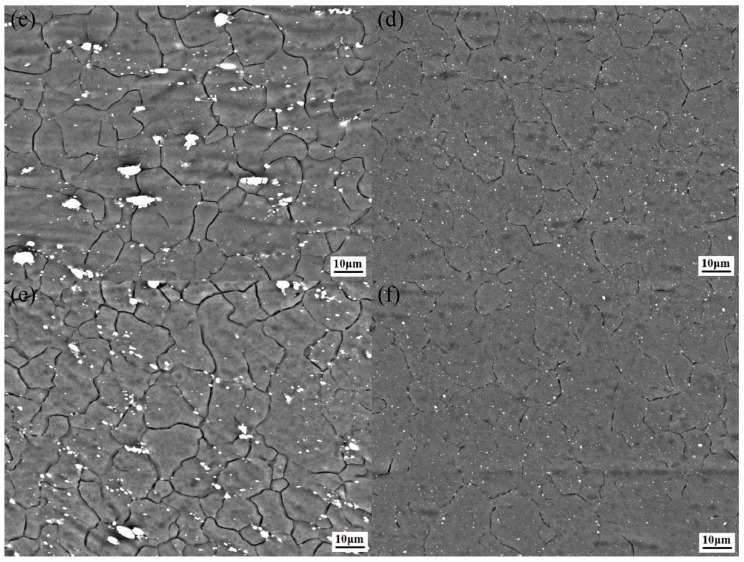
SEM images of different stir zone (SZ) regions in FSP Mg–6Sn–2Zn samples: (**a**) SZ-up region at 60 mm/min; (**b**) SZ-down region at 60 mm/min; (**c**) SZ-up region at 90 mm/min; (**d**) SZ-down region at 90 mm/min; (**e**) SZ-up region at 120 mm/min; and (**f**) SZ-down region at 120 mm/min.

Figure 4 shows SEM images of different SZ regions in FSP Mg–6Sn–2Zn samples. As shown in Figure 4, dynamic recrystallization (DRX) takes place during FSP in both SZ-up and SZ-down regions of the Mg–6Sn–2Zn alloy. Compared to the BM, coarse α-Mg grains are refined significantly in the SZ. Detailed statistics on average sizes of DRXed grains in each region were achieved by the following EBSD characterization. It was also found that FSP has an obvious influence on the size and relative content of the Mg_2_Sn phase in different SZ regions. As shown in Figure 4a,c,e, the eutectic Mg_2_Sn phase was mainly broken into coarse particles and distributed at the boundaries of DRXed grains. Meanwhile, some fine Mg_2_Sn particles were also observed within DRXed α-Mg grains. Different from SZ-up regions, only some fine Mg_2_Sn particles were observed within the α-Mg matrix in SZ-down regions (seen in Figure 4b,d,f). Compared to SZ-up regions, the relative content of the Mg_2_Sn phase in SZ-down regions decreased significantly. This result indicated that most of the Mg_2_Sn phase in SZ-down regions was dissolved into the matrix during FSP.

**Figure 5:**
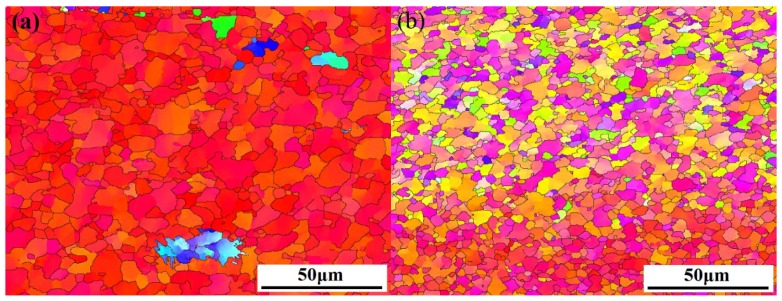

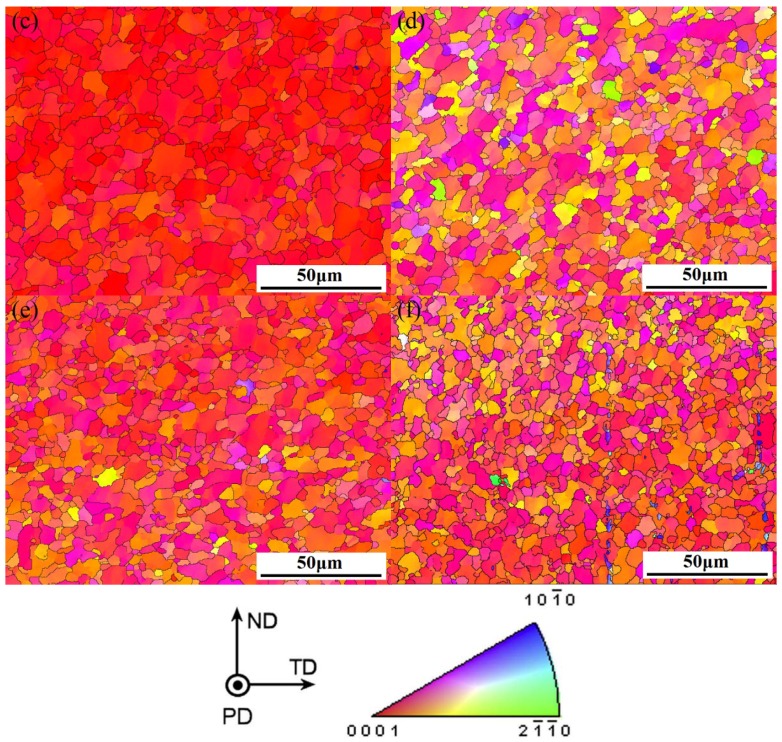
Inverse pole figure maps of different SZ regions in FSP Mg–6Sn–2Zn samples: (**a**) SZ-up region at 60 mm/min; (**b**) SZ-down region at 60 mm/min; (**c**) SZ-up region at 90 mm/min; (**d**) SZ-down region at 90 mm/min; (**e**) SZ-up region at 120 mm/min; and (**f**) SZ-down region at 120 mm/min.

Figure 5 shows inverse pole figure maps of different SZ regions in FSP Mg–6Sn–2Zn samples obtained by EBSD. Similar to the observation in Figure 4, the EBSD result confirms that complete DRX takes place in SZ regions of three FSP Mg–6Sn–2Zn samples. It was found in three FSP samples that DRXed grains in SZ-up regions were coarser than those in SZ-down regions. The shape of DRXed grains was irregular and the size of DRXed grains was uneven. Unlike the assumed random orientation in as-cast alloys, most of DRXed grains in FSP Mg–6Sn–2Zn samples exhibited an obviously preferred orientation.

**Figure 6:**
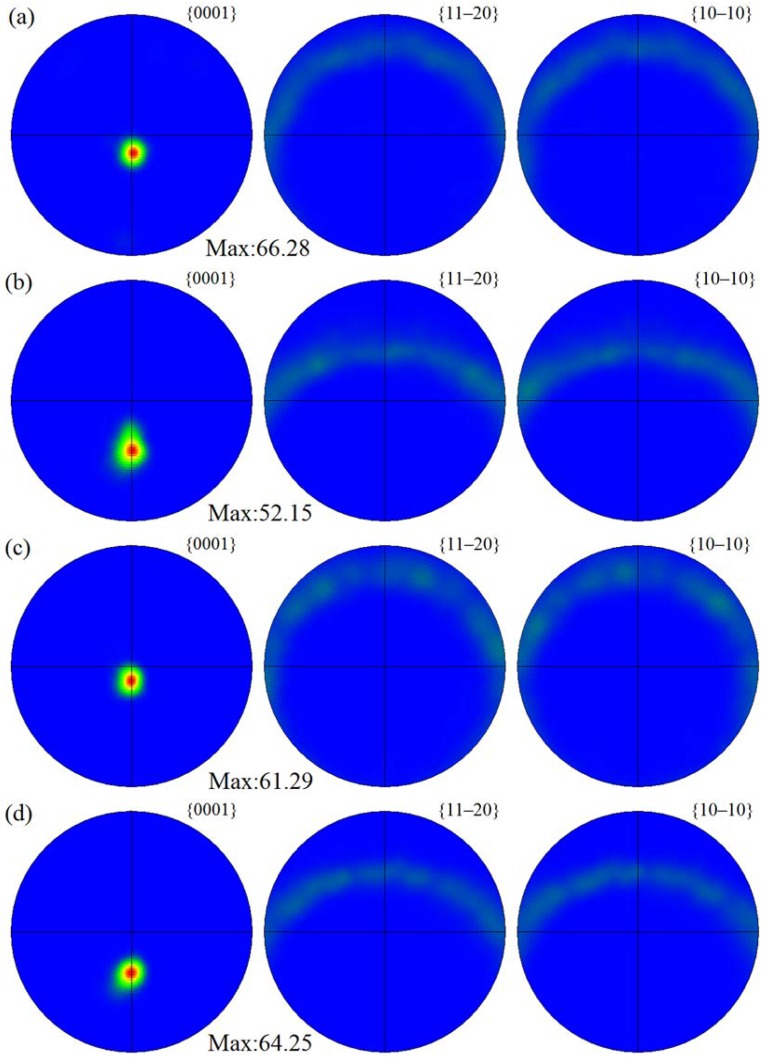

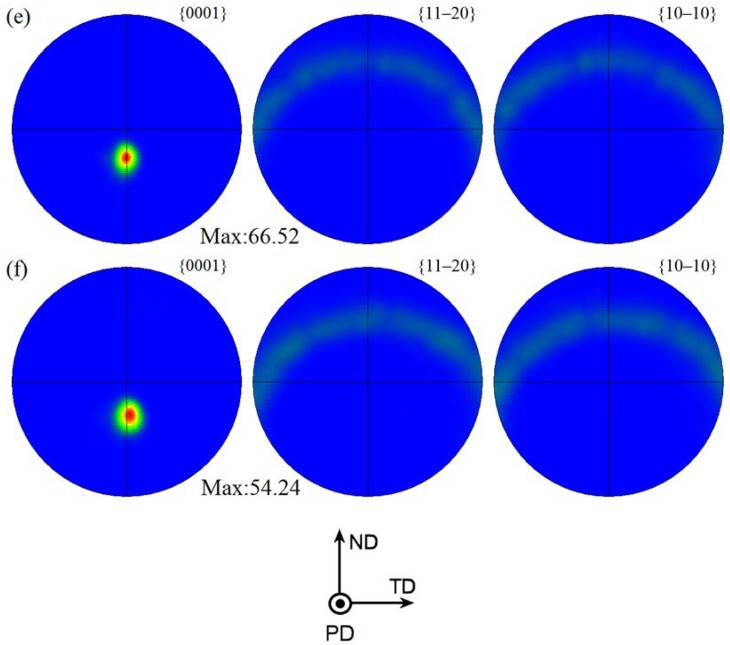
Pole figures of different SZ regions in FSP Mg–6Sn–2Zn samples: (**a**) SZ-up region at 60 mm/min; (**b**) SZ-down region at 60 mm/min; (**c**) SZ-up region at 90 mm/min; (**d**) SZ-down region at 90 mm/min; (**e**) SZ-up region at 120 mm/min; and (**f**) SZ-down region at 120 mm/min.

Figure 6 shows the corresponding pole figures of different SZ regions in FSP Mg–6Sn–2Zn samples obtained by EBSD. A strong {0001} basal texture was developed in SZ regions of Mg–6Sn–2Zn samples after FSP. A relatively high intensity of basal texture was achieved (fluctuating within the range of 52.2–66.5). Meanwhile, the intensity of {11–20} and {10–10} non-basal textures were relatively weak. Compared to SZ-up regions, the <0001> orientation in SZ-down regions was slightly tilted from PD to ND. As shown in Figure 6, with the increase of travel speed, there was little difference in the texture of different SZ regions.

**Figure 7:**
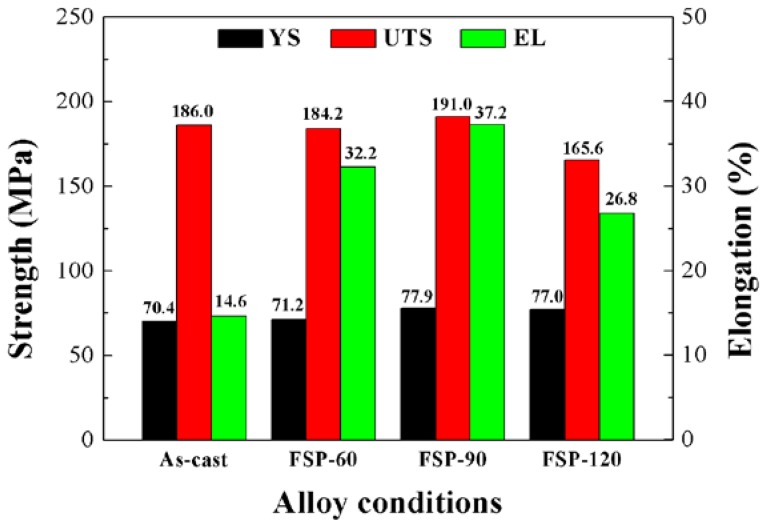
Mechanical properties of the as-cast and FSP Mg–6Sn–2Zn samples: As-cast represents the as-cast sample and FSP-60, FSP-90, and FSP-120 represent the FSP Mg–6Sn–2Zn samples with travel speeds of 60 mm/min, 90 mm/min, and 120 mm/min, respectively.

Figure 7 shows the tensile properties of as-cast and FSP Mg–6Sn–2Zn samples. The yield strength (YS), ultimate tensile strength (UTS), and elongation (EL) of as-cast Mg–6Sn–2Zn alloy were 70.4 MPa, 186.0 MPa, and 14.6%, respectively. After FSP, the EL of Mg–6Sn–2Zn samples was significantly improved. However, the enhancement in strength was not obvious. For FSP Mg–6Sn–2Zn samples, with the travel speed increase from 60 mm/min to 90 mm/min, the tensile properties were improved slightly. However, when the travel speed was further increased to 120 mm/min, both the UTS and EL of the FSP Mg–6Sn–2Zn sample decreased obviously. The FSP Mg–6Sn–2Zn sample with a travel speed of 90 mm/min exhibited the best comprehensive tensile properties and its YS, UTS, and EL were 77.9 MPa, 191.0 MPa, and 37.2%, respectively. Compared to the as-cast sample, the EL of the FSP Mg–6Sn–2Zn sample with a travel speed of 90 mm/min was improved by 155%.

## 4. Discussion

In this study, it was found that DRX takes place during FSP and results in a completely DRXed grain structure in the SZ of Mg–6Sn–2Zn samples. DRX and the corresponding grain refinement during FSP is generally attributed to the severe plastic deformation and thermal exposure. The severe plastic deformation is mainly due to the high-speed rotation of the stirring pin and the thermal exposure is due to the frictional heat between the shoulder and the top surface of the workpiece [19]. Chang et al. established a relationship between the material flow strain rate $\dot{\varepsilon }$, the average material flow rate *R_m_*, the effective radius *r_e_*, and the effective depth *L_e_* of the DRXed zone for FSP AZ31 alloy [20]:

(1) $$\overset{\cdot }{\varepsilon }=\frac{R_{m}\cdot 2\pi r_{e}}{L_{e}}$$

where the average material flow rate *R_m_* is about half of the rotation speed, the effective radius *r_e_* and the effective depth *L_e_* of DRXed zone are assumed to equal to about 0.78 of the observed zone boundary radius and depth. According to Equation (1), the strain rate is mainly influenced by the rotation speed. Due to the rotation lagging effect, the travel speed can influence the strain rate via the change of the effective radius *r_e_* and the effective depth *L_e_* of the DRXed zone. However, compared to the rotation speed, the effect of the travel speed on the strain rate is slight. In this study, the strain rate of the FSP Mg–6Sn–2Zn sample with a travel speed of 60 mm/min was calculated to be about 96.8 s^−1^. As the rotation speed was kept constant and the macrostructural characteristics of three FSP samples were almost the same, it is reasonable to assume that the strain rate of FSP in this study was about 10^2^ s^−1^. The typical recrystallization temperatures for Mg alloys were 0.5—0.7 *T_m_*. During FSP, the temperature of Mg alloys could reach the temperature range. Under the combined effect of high strain rate and elevated temperature, complete DRX and significant grain refinement were achieved in the FSP Mg–6Sn–2Zn samples.

**Figure 8:**
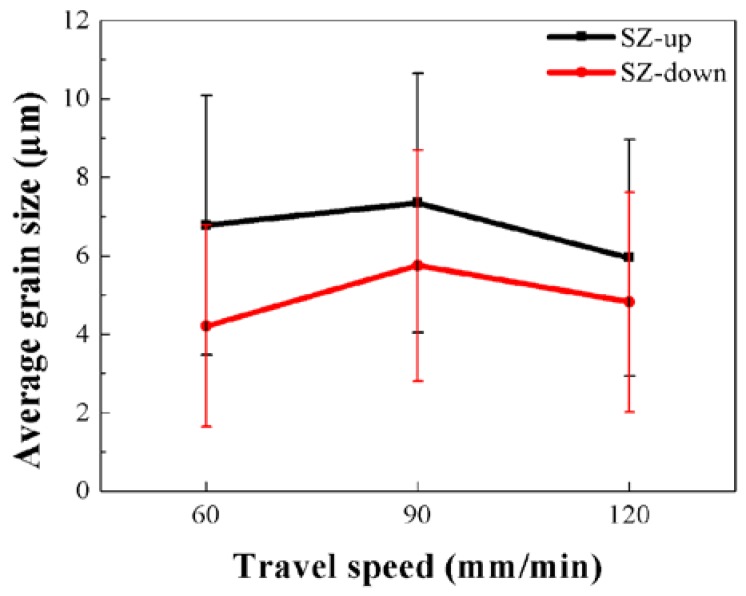
Effect of the travel speed on the average size of dynamic recrystallized (DRXed) grains in different SZ regions of the FSP Mg–6Sn–2Zn samples.

When FSP is conducted at a constant rotation speed, increase of travel speed leads to the decrease of heat input and the reduced heat input is beneficial to grain refinement. Figure 8 shows the quantitative analysis of the effect of the travel speed on the average size of DRXed grains in different SZ regions. According to the result in Figure 8, when the travel speed increased from 90 mm/min to 120 mm/min, the average size of DRXed grains in both SZ-up and SZ-down regions decreased. However, when the travel speed increased from 60 mm/min to 90 mm/min, DRXed grains in both SZ-up and SZ-down regions were coarsened. Li et al. also found that the increase of travel speed did not lead to continuous grain refinement in FSP Mg–Y–Nd–Zr alloy [21]. It was also found that, in all three FSP samples, DRXed grains in SZ-up regions were coarser than those in SZ-down regions. Since the frictional heat is generated on the top surface of the SZ, SZ-up regions, which are close to the top surface, are subjected to greater thermal effect than SZ-down regions. For example, Han et al. studied the temperature distribution during FSP of an Mg–Nd–Zn–Zr alloy and found that the peak temperature occurred in the top portions of the SZ [22]. On the contrary, SZ-down regions undergo more intense plastic deformation than SZ-up regions during FSP, since SZ-up regions are deformed under the combined effect of shear deformation by the rotating pin and compressive deformation by the shoulder [23]. The final size of DRXed grains was determined by the competition between the grain refinement caused by severe plastic deformation and the coarsening caused by thermal exposure. Since the DRXed grains in SZ-up regions were subjected to greater thermal exposure and weaker plastic deformation than SZ-down regions, the DRXed grains in SZ-up regions were coarser than SZ-down regions after FSP.

**Figure 9:**
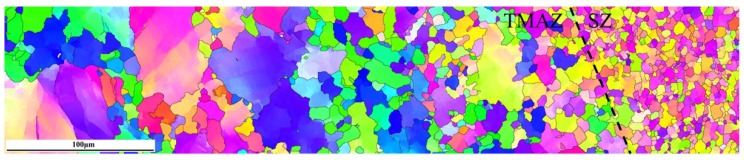

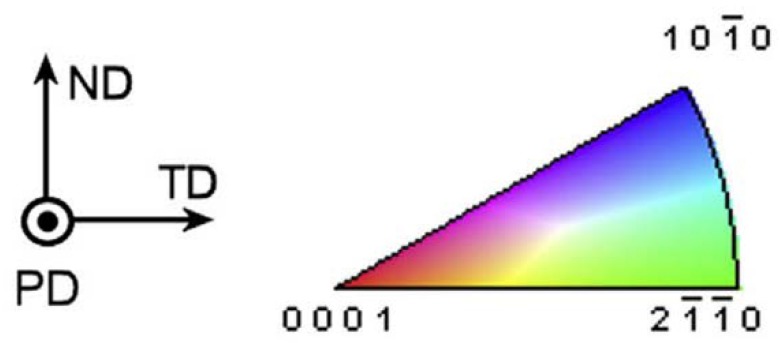
Inverse pole figure maps of region A in the Mg–6Sn–2Zn sample with a travel speed of 60 mm/min.

**Figure 10:**
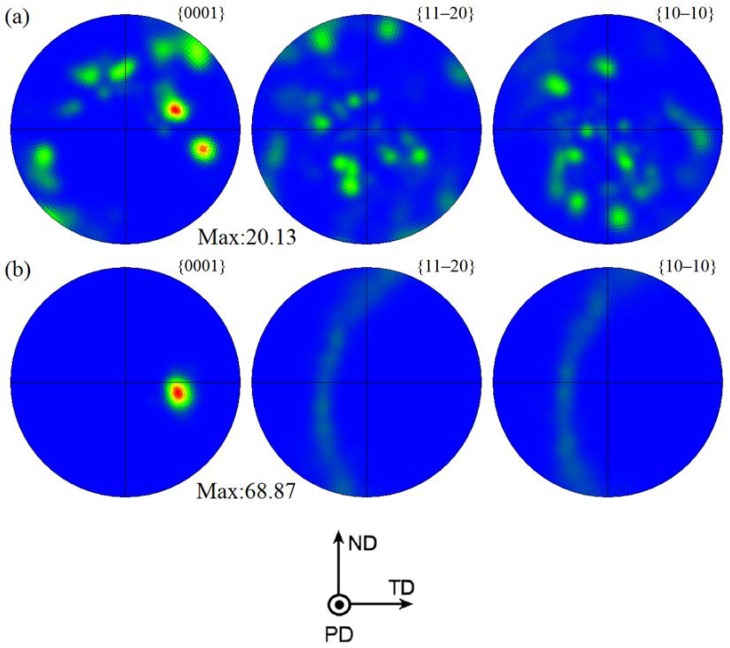
Pole figures of the thermo-mechanically affected zone (TMAZ) and SZ in region A of the Mg–6Sn–2Zn sample with a travel speed of 60 mm/min: (**a**) TMAZ and (**b**) SZ.

As seen in Figure 6, FSP introduces basal texture in the SZ regions of Mg–6Sn–2Zn samples, which was also observed in several FSP Mg alloys [24,25,26]. The intensity of {0001} basal texture reached as high as 52.2–66.5. The results in Figure 5 and Figure 6 confirm that, due to the intensive shear deformation of the pin, the basal slip planes in SZ were rotated to be parallel to the pin surface. To further release the texture evolution of the Mg–6Sn–2Zn alloy after FSP, an EBSD observation with a relatively large area was performed in the Mg–6Sn–2Zn sample with a travel speed of 60 mm/min (the observed area is labeled as region A in Figure 3). As shown in Figure 9, the orientation map can be roughly divided into two parts, i.e., the TMAZ and the SZ. Since the observed area is in the AS, the transition from the TMAZ to SZ is sharp. It can be observed form Figure 8 that the size and orientation of grains are different from each other in the TMAZ and SZ. The TMAZ is a mixture of coarse un-DRXed and DRXed grains, which is always observed in FSP samples. Compared to the SZ, the orientation of grains in the TMAZ is relatively random. Complete DRX is observed in the SZ and the grains are further refined. Figure 10 shows the pole figures of the TMAZ and SZ in Figure 9. A strong {0001} texture was formed in the SZ. From the TMAZ to SZ, the intensity of the {0001} texture increased from 20.13 to 68.87. Compared to the pole figures in Figure 6, it was found that the <0001> orientation in Figure 10b is tilted from PD to TD, which also confirms that the {0001} plane is roughly parallel to the surface of the pin. Such texture characteristics were also found in previous studies on FSP Mg–Al–Zn (AZ) and Mg–Zn–Zr (ZK) alloy series [27,28].

**Figure 11:**
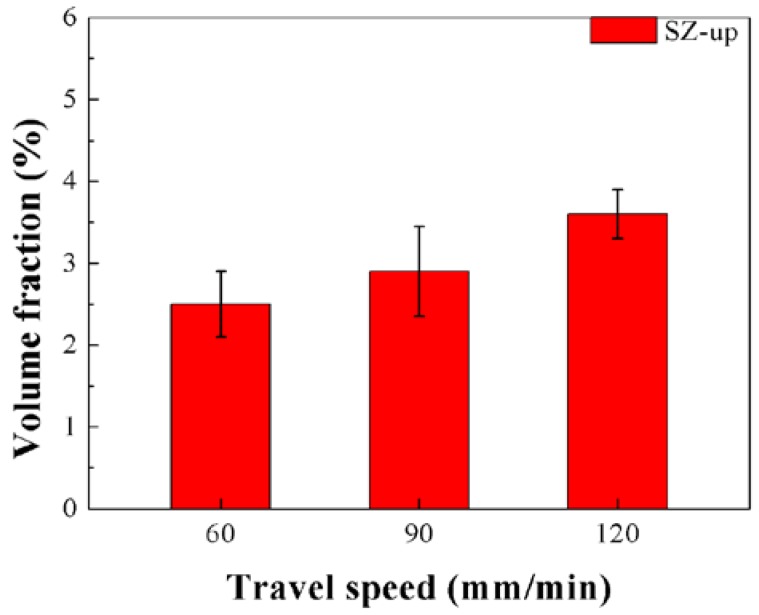
Effect of the travel speed on the relative content of the remaining Mg_2_Sn phase in SZ-up regions of the FSP Mg–6Sn–2Zn samples.

Pan et al. found in FSW Mg–Al–Sn extruded plates that the β-Mg_17_Al_12_ phase with a low melting point dissolved while the Mg_2_Sn phase remained after FSW [29]. However, in this study, a partial dissolution of the Mg_2_Sn phase during FSP was observed. Figure 11 shows the effect of the travel speed of remaining Mg_2_Sn phase in SZ-up regions of the FSP Mg–6Sn–2Zn samples. Since the remaining Mg_2_Sn phases in SZ-down regions were all lower than 0.3 vol.%, the data is omitted in Figure 11. As seen in Figure 11, according to the result of the quantitative metallurgical analysis of the volume fraction of the Mg_2_Sn phase, with the increase of travel speed, the relative content of the remaining Mg_2_Sn phase in SZ-up regions increased gradually. The relative content of the Mg_2_Sn phase in the BM was about 4.7 vol.%. It was concluded that the relative content of the Mg_2_Sn phase dissolved during FSP was reduced with the increase of travel speed, which is in accordance with the reduction of heat input. As shown in Figure 4, although the peak temperatures of SZ-up regions reached during FSP were higher than those achieved in SZ-down regions, coarse broken Mg_2_Sn particles were observed in SZ-up regions, while only fine Mg_2_Sn particles were observed in the α-Mg matrix in SZ-down regions. It was also found that, in FSP Mg–Gd–Y–Nd–Zr alloy, second phase particles were observed in SZ-up regions rather than SZ-down regions [23]. These results indicated that the temperature is not the main influence factor in the dissolution of the Mg_2_Sn phase during FSP. In SZ-down regions, the Mg_2_Sn phase is broken into fine particles and its dissolution becomes easy due to the rapidly increased surface area. In SZ-up regions, although the temperature is higher than SZ-down regions, the broken Mg_2_Sn particles are coarse. Since the diffusion rate of Sn element in the α-Mg matrix is low and the duration time of FSP is short, the acceleration of the dissolution of the Mg_2_Sn phase caused by elevated temperatures is not obvious.

The results of tensile properties of FSP Mg–6Sn–2Zn samples in Figure 8 show that FSP improved the ductility of the Mg–6Sn–2Zn samples obviously, while the improvement in strength was negligible. Since DRX takes place during FSP and significant grain refinement is achieved, according to classic Hall-Petch equation, the YS of FSP Mg–6Sn–2Zn samples should also be improved obviously. The combined enhancement of ductility and strength by FSP has been achieved in some Mg alloys [30,31,32]. However, there are also studies that have reported that FSP could only improve the ductility of Mg alloys. For example, Woo et al. found in FSP AZ31 alloy that FSP led to an increase in elongation and a decrease in strength [33]. The similar phenomenon was also found by Vargas et al. in FSP of Mg–Zn–Ca–Zr alloy [19]. In fact, the tensile strength of FSP Mg alloys is influenced by several factors, including grain refinement, texture modification, and the breaking and dissolution of second phases. The strong basal texture indicates the reorientation of the basal slip system, which reduces the deformation resistance and leads to a relatively low strength. For as-cast Mg alloys, the coarse second phases can partially retard the dislocation slip during plastic deformation. During the heat treatment of Mg alloys, the dissolution of second phases via solid solution treatment usually leads to improved EL but reduced YS. For FSP Mg alloys, the breaking up and dissolution of second phases may have a similar effect. For FSP Mg–6Sn–2Zn samples, although FSP induced DRX and corresponding grain refinement, the formation of strong basal texture and breaking up and dissolution of the Mg_2_Sn phase may be harmful to their strength. Therefore, in this study, the final strength of the FSP Mg–6Sn–2Zn samples was little improved when compared to as-cast state. As for the ductility of FSP Mg–6Sn–2Zn samples, the influence factors including grain refinement, texture modification, and the breaking up and dissolution of second phases were all beneficial to ductility. Therefore, significant improvement in elongation was observed in the FSP Mg–6Sn–2Zn samples with different travel speeds, which is always observed in other FSP Mg alloys as well. For the FSP Mg–6Sn–2Zn samples with different travel speeds, since the strength is not only determined by grain size, the samples with a small grain size did not exhibit high strength.

## 5. Conclusions

In this study, as-cast Mg–6Sn–2Zn alloy was prepared by gravity casting and then subjected to FSP with different travel speeds. The microstructure and tensile properties of the FSP Mg–6Sn–2Zn samples were investigated. Our main conclusions are listed as follows.

(1) Complete DRX took place in the SZ of FSP Mg–6Sn–2Zn samples, and α-Mg grains were significantly refined. DRXed grains in SZ-up regions were coarser than those in SZ-down regions. The Mg_2_Sn phase in the as-cast Mg–6Sn–2Zn alloy was broken and partially dissolved. Coarse broken Mg_2_Sn particles were observed in SZ-up regions, while only fine Mg_2_Sn particles were observed in SZ-down regions. The difference in microstructure of different SZ regions was due to the combined effect of severe plastic deformation and thermal exposure.

(2) Most of the DRXed grains in the FSP Mg–6Sn–2Zn samples exhibited obviously preferred orientation, and strong {0001} basal texture was developed in SZ-up and SZ-down regions. The intensity of the {0001} basal texture reached as high as 52.2–66.5. With the increase of travel speed, little difference was found in the texture of different SZ regions.

(3) Compared to the as-cast Mg–6Sn–2Zn alloy, the EL of the FSP Mg–6Sn–2Zn samples was improved obviously. However, the improvement in strength was negligible. For the FSP Mg–6Sn–2Zn samples, although FSP induced DRX and corresponding grain refinement, the formation of a strong {0001} basal texture and the breaking up and dissolution of the Mg_2_Sn phase may be harmful to their strength. The influence factors, including grain refinement, texture modification, and the breaking up and dissolution of the Mg_2_Sn phase, were all beneficial to the improvement fo the EL.
